# Genomic Analysis of *Enterococcus* spp. Isolated From a Wastewater Treatment Plant and Its Associated Waters in Umgungundlovu District, South Africa

**DOI:** 10.3389/fmicb.2021.648454

**Published:** 2021-06-14

**Authors:** Joshua Mbanga, Daniel G. Amoako, Akebe L. K. Abia, Mushal Allam, Arshad Ismail, Sabiha Y. Essack

**Affiliations:** ^1^Antimicrobial Research Unit, College of Health Sciences, University of Kwazulu-Natal, Durban, South Africa; ^2^Department of Applied Biology and Biochemistry, National University of Science and Technology, Bulawayo, Zimbabwe; ^3^Sequencing Core Facility, National Institute for Communicable Diseases, National Health Laboratory Service, Johannesburg, South Africa

**Keywords:** *Enterococcus* spp., whole-genome sequencing, wastewater treatment plant, antibiotic resistance, South Africa

## Abstract

We investigated the antibiotic resistome, mobilome, virulome, and phylogenomic lineages of *Enterococcus* spp. obtained from a wastewater treatment plant and its associated waters using whole-genome sequencing (WGS) and bioinformatics tools. The whole genomes of *Enterococcus* isolates including *Enterococcus faecalis* (*n* = 4), *Enterococcus faecium* (*n* = 5), *Enterococcus hirae* (*n* = 2), and *Enterococcus durans* (*n* = 1) with similar resistance patterns from different sampling sites and time points were sequenced on an Illumina MiSeq machine. Multilocus sequence typing (MLST) analysis revealed two *E. faecalis* isolates that had a common sequence type ST179; the rest had unique sequence types ST841, and ST300. The *E. faecium* genomes belonged to 3 sequence types, ST94 (*n* = 2), ST361 (*n* = 2), and ST1096 (*n* = 1). Detected resistance genes included those encoding tetracycline [*tet*(S), *tet*(M), and *tet(L*)], and macrolides [*msr*(C), *msr(D)*, *erm(B)*, and *mef*(A)] resistance. Antibiotic resistance genes were associated with insertion sequences (IS6, ISL3, and IS982), and transposons (Tn3 and Tn6000). The *tet(M*) resistance gene was consistently found associated with a conjugative transposon protein (TcpC). A total of 20 different virulence genes were identified in *E. faecalis* and *E. faecium* including those encoding for sex pheromones (*cCF10, cOB1, cad*, and *came*), adhesion (*ace, SrtA, ebpA, ebpC*, and *efaAfs*), and cell invasion (*hylA* and *hylB*). Several virulence genes were associated with the insertion sequence IS256. No virulence genes were detected in *E. hirae* and *E. durans.* Phylogenetic analysis revealed that all *Enterococcus* spp. isolates were more closely related to animal and environmental isolates than clinical isolates. *Enterococcus* spp. with a diverse range of resistance and virulence genes as well as associated mobile genetic elements (MGEs) exist in the wastewater environment in South Africa.

## Introduction

The efficiency of wastewater treatment plants (WWTPs) is critical to preventing the spread of antibiotic resistant bacteria (ARB) and antibiotic resistance genes (ARGs) into the environment (Karkman et al., 2017; Alexander et al., 2020). Although AMR surveillance in clinical settings and animals is well established in most developed and some developing countries, surveillance in the environment still lags behind (Huijbers et al., 2019). The emergence of ARB and ARGs in the water environment has become an important environmental health issue (Conte et al., 2017; Karkman et al., 2017; Alexander et al., 2020). Dissemination of ARGs is thought to occur in the environment mainly through the transfer of mobile genetic elements (MGEs) such as plasmids, transposons, integrons, gene cassettes, Integrative and conjugative elements (ICE), and insertion sequence common regions between bacterial species (Sanderson et al., 2020). The selection pressure in a given environment is crucial as it influences the spread and accumulation of ARGs some of which may be novel (Bengtsson-Palme et al., 2018). The risk of transfer of ARGs to pathogens increases in environments with a high fecal load and associated fecal bacteria (Huijbers et al., 2019).

*Enterococcus* species are Gram positive non-sporulating organisms that mainly exist as commensals in the intestinal flora of healthy animals and humans. They can thus be excreted into environmental sources including soil and surface water as fecal matter and are thus commonly used as indicator organisms in water environments (Berendonk et al., 2013; Karkman et al., 2018). Some like *faecalis* and *Enterococcus faecium* are opportunistic pathogens whilst other species such as *Enterococcus hirae* and *Enterococcus durans* are rarely pathogenic in humans (Bourafa et al., 2015; Ryu et al., 2019). *Enterococcus* spp. can easily acquire and disseminate resistance determinants (Medeiros et al., 2014) making them suitable for antibiotic resistance surveillance studies.

Whole-genome sequencing (WGS) is a highly discriminatory technique for studying bacterial species, including Enterococci. However, very few studies have used WGS to study environmental enterococcal isolates (Sanderson et al., 2020; Zaheer et al., 2020). The application of WGS to antibiotic resistance surveillance remains largely confined to clinical and animal settings, with very little attention given to the environment (Hendriksen et al., 2019; Su et al., 2019; WHO, 2020). There is therefore a paucity of data on the role that genomic surveillance plays in understanding the environmental dimensions of antibiotic resistance, particularly in Africa.

In this study, we investigated the antibiotic resistome, mobilome, virulome, and phylogenomic lineages of *Enterococcus* spp. obtained from a WWTP and its associated waters. Additionally, we assayed the role of the water environment in the dissemination of multi-drug resistant *Enterococcus* spp. which could be of clinical or veterinary importance.

## Materials and Methods

### Ethical Consideration

Ethical approval was received from the Biomedical Research Ethics Committee (Reference: BCA444/16) of the University of KwaZulu-Natal. Permission to collect water samples was sought and granted by uMgeni Water which owns and operates the investigated WWTP.

### Study Site

Manual grab water samples were collected in sterile 500-mL containers from the influent (29°36′3.70″S 30°25′41.71″E), final effluent (29°35′49.97″S 30°26′19.74″E) of a major WWTP as well as upstream (29°36′10.73″S 30°25′29.97″E) and downstream (29°36′27.54″S 30°27′0.76″E) of its associated receiving water body in uMgungundlovu District, KwaZulu-Natal, South Africa.

The WWTP is the largest in Pietermaritzburg, the provincial capital of KwaZulu-Natal in South Africa. The WWTP discharges its final effluent into the uMsunduzi river, a key water source for domestic, agricultural, and recreational purposes to inhabitants of the several informal settlements along its banks (Moodley et al., 2016).

### Bacterial Isolates

*Enterococcus* spp. were isolated from water samples collected fortnightly over 7 months (May 2018 to November 2018).

Putative identification was accomplished during enumeration using the Enterolert^®^/Quanti-Tray^®^ 2000 system followed by phenotypic confirmation on Bile Aesculin Azide agar (Merck, Germany) or Slanetz and Bartley agar (Merck, Germany). Samples from, upstream and downstream river water as well as final effluent were diluted 1 mL in 100 mL (0.01 dilution) while the influent with its higher bacterial load was o diluted by 0.05 mL in 100 mL (0.005 dilution) using sterile water. A volume of 100 mL of each sample was analyzed using the Enterolert^®^ Quanti-Tray^®^ 2000 system (IDEXX Laboratories (Pty.) Ltd., Johannesburg, South Africa). *Enterococcus* spp. were obtained from positive quanti-trays, sub-cultured on Bile Aesculin Azide or Slanetz and Bartley agar and incubated at 41°C for 24–48 h. At least ten distinct colonies representing each sampling site were randomly selected from the Bile Aesculin Azide or Slanetz and Bartley agar and further sub-cultured onto the same media, respectively, to obtain pure colonies. Molecular confirmation of *Enterococcus* spp. was done using real-time polymerase chain reaction (rtPCR) of the *tuf* (Elongation factor *tu*) gene (Ke et al., 1999).

Antibiotic susceptibility determination was undertaken using the Kirby-Bauer method on a panel of sixteen commercial antibiotic discs which included: chloramphenicol (CHL, 30 μg), tetracycline (TET, 30 μg), ampicillin (AMP, 10 μg), nitrofurantoin (NIT, 300 μg), ciprofloxacin (CIP, 5 μg), levofloxacin (LVX, 5 μg), imipenem (IPM, 10 μg), linezolid (LZD, 30 μg), erythromycin (E, 15 μg), quinupristin–dalfopristin (Q–D, 15 μg) against *E. faecium* only, tigecycline (TGC, 15 μg), trimethoprim-sulfamethoxazole (SXT, 25 μg), vancomycin (VAN, 30 μg) and teicoplanin (TEC, 30 μg). Detection of high-level aminoglycoside resistance was ascertained using gentamicin (GEN, 120 μg) and streptomycin (STR, 300 μg) discs. Inhibition zones were measured, and the results were interpreted using the European Committee on Antimicrobial Testing (EUCAST) breakpoint tables (EUCAST, 2020). Breakpoints for chloramphenicol, tetracycline, erythromycin, linezolid, nitrofurantoin, vancomycin, and gentamicin were obtained from the Clinical and Laboratory Standards Institute (CLSI) interpretative charts (CLSI, 2020). *Enterococcus faecalis* ATCC 29212 was used for quality control.

### Whole-Genome Sequencing and Analysis

Twelve MDR *Enterococcus* spp. isolates with similar antibiograms obtained from all four sampled sites were selected for WGS. Genomic DNA was extracted using the GenElute Bacterial Genomic DNA kit (Sigma Aldrich, St. Louis, United States) followed by quantification using the 260/280 nm wavelength on a Nanodrop 8000 (Thermo Scientific Waltham, MA, United States). Library preparation was done using the Nextera XT DNA Library Preparation Kit (Illumina, San Diego, CA, United States). WGS was undertaken using an Illumina MiSeq machine (Illumina, San Diego, CA, United States). The raw reads were quality trimming using Sickle v1.33^1^ and assembled spontaneously using the SPAdes v3.6.2 assembler. All contiguous sequences were subsequently submitted to GenBank and assigned accession numbers under Bio project PRJNA609064 (Supplementary Table 1).

The assembled genomes were analyzed for MLST sequence types on the MLST 1.8 database (Larsen et al., 2012) hosted by the Centre for Genomic Epidemiology (CGE)^2^. Acquired antimicrobial resistance genes and chromosomal point mutations including the DNA gyrase *gyr*A and *parC* genes (quinolone resistance) and the pbp5 gene (ampicillin resistance) were annotated using ResFinder^3^ set at default threshold ID (90%) and minimum length (60%) values. Plasmid replicons types were identified using PlasmidFinder 2.1 on the CGE website^4^. Virulence genes were determined using VirulenceFinder 2.0 on the CGE website^5^.

The assembled genomes were further analyzed for MGEs, including insertion sequences, using ISFinder^6^ (Siguier, 2006), and intact prophages using PHASTER^7^ (Zhou et al., 2011; Arndt et al., 2016). ICE and putative integrative and mobilisable elements (IME) were identified using the ICEberg database^8^. RAST SEEDVIEWER^9^ was also used to annotate and identify the genomes with integrons, and transposons. The synteny and genetic environment of ARGs and associated MGEs were investigated using the general feature format (GFF3) files from GenBank. The genetic environment of virulence genes detected in the study were also determined using a similar approach. The GFF files were imported into Geneious prime 2020.2^10^ for analysis.

#### Phylogenetic Reconstruction

Whole-genome sequences of the *E. faecalis* and *E. faecium* isolates were compared with isolates curated from the PATRIC website^11^ from different African countries including South Africa. The genomes of *E. hirae* and *E. durans* isolates were compared to those of isolates belonging to the respective species curated from the PATRIC website from different countries across the world as there were no/few entries from Africa. Whole-genome sequences of all isolates were uploaded and analyzed on the CSI Phylogeny 1.4 pipeline^12^ that recognizes, screens, and validates the location of single nucleotide polymorphisms (SNPs) before deducing a phylogeny based on the concatenated alignment of the high-quality SNPs. SNPs were identified from the alignments using the mpileup module in SAMTools version 0.1.18 (Li et al., 2009). Selection of SNPs was based on default parameters in CSI Phylogeny (Kaas et al., 2014). The following reference genomes were used for each alignment; *E. faecalis*, (*E. faecalis* V583), *E. faecium* (*E. faecium* DO), *E. hirae* (*E. hirae* ATCC 9790), and *E. durans* (*E. durans* ATCC 6056). The phylogenetic tree was constructed using FastTree (Price et al., 2010). The generated phylogenetic trees were viewed, annotated, and edited using the Iterative Tree of Life (iTOL)^13^.

## Results

### Isolate Source and Antibiotic Susceptibility Patterns

A total of 579 *Enterococcus spp.* isolates were obtained from the different sampling points. Of these, 12 isolates were selected for WGS, distributed as follows: three isolates from the upstream site of the WWTP along the receiving river, four from the downstream site, three from the raw influent, and two were from the final effluent of the WWTP (Supplementary Table 1). Selected isolates consisted of *E. faecalis* (4 isolates), *E. faecium* (5), *E. hirae* (2), and *E. durans* (1) (Supplementary Table 1).

The resistance patterns displayed by the selected isolates from different time points and sampling sites are shown in Table 1. The resistance profile TET-SXT-STR was found in two isolates, one *E. faecalis* obtained from raw influent and one *E. durans* isolate from the influent. Two *E. faecalis* isolates from downstream and upstream sites had the same resistance profile ERY-TEC-TET-SXT, other resistance profiles shared by at least two isolates from different sites included *E. faecium* (QD-TET-SXT, QD-TET-SXT-STR), and *E. hirae* (NIT-SXT-STR) isolates. The resistance profiles TEC-QD-TET-SXT, and ERY-TEC-LZD-TET-SXT-GEN-STR were unique to individual isolates (Table 1).

**TABLE 1:** Distribution of antibiotic resistance genes and mobile genetic elements in environmental *Enterococcus* spp.

Isolate ID (MLST)	Resistance pattern	Date of isolation	Point of isolation	Detected ARG	Insertion sequences	Intact prophages	Putative ICE	Putative IME	Plasmid replicon type
***E. faecalis***									
°D21 (ST 179)	ERY-TEC-TET-SXT	Jun 12, 2018	Downstream	*dfrG, erm(B), lsa(A), tet(M), aac(6*′*)-aph(2*″*), ant(6)-Ia, aph(3*′*)-III*	ISEfa10, ISEfa11, ISEfa5, ISLsa2	No intact prophage	T4SS type ICE	None detected	repUS43
°U84 (ST 300)	ERY-TEC-TET-SXT	Sep 11, 2018	Upstream	*dfrG, erm(B), lsa(A), tet(L), tet(M)*	ISCac2, ISCysp18, TnBth2, ISMspa1	Entero_phiFL1AEntero_ phiFL3A	None detected	1	repUS43 rep9b
°IN127 (ST 179)	ERY-TEC-LZD-TET-SXT-GEN-STR	Oct 23, 2018	Influent	*erm(B), lsa(A), tet(M)*	ISEfa10, ISEfa11, ISEfa5, ISLsa2	Entero_phiFL1A Entero_phiFL3A	None detected	1	repUS43 rep9c
°IN133 (ST 841)	TET-SXT-STR	Nov 6, 2018	Influent	*lsa(A), msr(C), tet(M), aac(6*′*)-Ii*	ISMspa1, ISLar7, ISArch1, ISBsp1	Lister_LP_101	T4SS type ICE	2	repUS15 repUS43
***E. faecium***									
°E21 (ST 1096)	TEC-QD-TET-SXT	Jun 12, 2018	Effluent	*msr(C), tet(M)*	ISEfa10, ISSpn11, ISSmu1, ISLgar1	Lister_B025	T4SS type ICE	1	No hits
°IN91 (ST 361)	QD-TET-SXT	Sep 11, 2018	Influent	*msr(C), tet(M)*	ISGaba2, ISFnu4, ISFnu3, MICBce1	Lister_B025, Bacill_BCJA1c	None detected	1	rep29, repUS43, repUS15
°D95 (ST 94)	QD-TET-SXT-STR	Sep 11, 2018	Downstream	*msr(C), tet(M)*	IS1485, ISLgar4, ISS1W, IS1216V	Entero_vB_IME197	T4SS type ICE	None detected	repUS43
°D98 (ST 94)	QD-TET-SXT	Sep 11, 2018	Downstream	*msr(C), tet(M)*	IS1485, ISEfa12, ISEfa8, ISSag12	Entero_vB_IME197	T4SS type ICE	None detected	repUS43, repUS15
°U129 (ST 361)	QD-TET-SXT-STR	Nov 6, 2018	Upstream	*lsa(A), msr(C), tet(M)*	ISPye7, ISShes9, ISBth14, ISFnu2	Entero_EFC_1	T4SS type ICE (3)	None detected	repUS24, repUS15, repUS43
***E. hirae***									
°D76	NIT-SXT-STR	Aug 14, 2018	Downstream	*aac(6*′*)-Iid*	ISEfa11, ISEfa5, IS1251, ISEfa10	No intact prophage	None detected	None detected	No hits
°U73	NIT-SXT-STR	Aug 28, 2018	Upstream	*aac(6*′*)-Iid*	ISEfa11, ISEfa5, IS1251, ISEfa12	No intact prophage	None detected	None detected	No hits
***E. durans***									
°E115	TET-SXT-STR	Oct 9, 2018	Effluent	*dfrG*, *mef(A), msr(D), tet(S), tet(M), aac(6*′*)-Iih,ant(6)-Ia*	ISEfa11, ISEfa5, ISSsu4, ISDha13	Bacill_BCJA1c Entero_phiFL1A	T4SS type ICE (3)	None detected	repUS15repUS1

*TET, tetracycline; NIT, nitrofurantoin; Q–D, quinupristin–dalfopristin; GEN, gentamicin; STR, streptomycin; LZD, Linezolid; ERY, erythromycin; SXT, trimethoprim-sulfamethoxazole; TEC, teicoplanin.*

### Genome Characteristics

The genome and assembly characteristics of the *Enterococcus* spp. sequences are presented in Supplementary Table 1. The total assembled genome size ranged from 2.5–3.2 MB, the GC content ranged from 36.6–38.4, the N50, L50; the total number of contigs are also shown in Supplementary Table 1.

### Antibiotic Resistance Genes

Several ARGs were present in the isolates, with each isolate harboring at least one ARG (Table 1). Most of the isolates belonging to all the sub-species harboured macrolides/streptogramins/lincosamides resistance genes *lsa(A)*, *msr(C)*, *msr(D)*, *erm(B)*, and *mef(A)*. Other ARGs included the tetracycline resistance [*tet*(S), *tet*(M), and *tet(L)*], aminoglycoside resistance [*aac(6*′*)-aph(2″), ant(6)-Ia, aph(3*′*)-III, aac(6*′*)-Iid, aac(6*′*)-Iih*], and trimethoprim resistance (*dfr*G) gene (Table 1). In *E. faecalis* macrolide resistance was mediated by the *erm(B)* gene – two isolates from the influent (IN127, ST179), and downstream (D21, ST179) sites had the *erm (B)*, *isa(A)*, and *tet(M)* genes in common. Tetracycline resistance was mediated mainly by the *tet(M)* gene in all the TET resistant 10/12 (83.3%) isolates except for one *E. faecalis* isolate (U84 ST300) from the upstream site that had *tet(M)* and *tet(L)*, as well as an *E. durans*, isolate (E115) from the effluent that had *tet(M)* and *tet(S)*. In the *E. faecium* isolates resistance genes could not be linked to sequence type or source of isolation as all the isolates had the *msr(C)*, and *tet(M)* genes in common (Table 1).

The quinolone resistance determinant regions (QRDRs) of the DNA gyrase (*gyr*A) and DNA topoisomerase IV genes (*par*C), were assayed for point mutations in all isolates. The *gyr*A (I259L^∗^, I306V^∗^, N708D^∗^, D759N^∗^, A811V^∗^, G819A^∗^, S820T^∗^, N708D^∗^), and *parC* (I699V^∗^, E707D^∗^, L773I^∗^) showed putatively novel mutations that were not linked to phenotypic resistance (Table 2). Only *E. faecium* isolates harboured mutations in all the assayed genes (Table 2). Point mutations in the *pbp5* gene which encodes ampicillin resistance were mostly putatively novel mutations (Table 2). No mutations were found in *E. faecalis*, *E. hirae*, and *E. durans*.

**TABLE 2:** Point mutation in the *gyrA*, *parC* (quinolone resistance), and *pbp5* (ampicillin resistance) genes in environmental *Enterococcus* spp.

Isolate ID	Pbp5	gyrA	parC
***E. faecium***			
°IN91	V24A, S27G, K144Q, K2E*, T25A*, S39T*, A73T*, R347C*	I259L*, I306V*, N708D*, D759N*, A811V*, G819A*, S820T*	I699V*, E707D*, L773I*
	R390C*, R474G*, Y475C*, K492Q*, K501E*, L573I*, S622N*		
	E646K*, K647E*, V684A*, T25A*, S39T*, D644N*		
°D95	V24A, S27G, K144Q, T324A, T25A*, S39T*, A73T*, D644N*	I259L*, I306V*, N708D*, D759N*, A811V*, G819A*, S820T*	I699V*, E707D*, L773I*
°D98	V24A, S27G, K144Q, T324A, T25A*, S39T*, A73T*, D644N*	I259L*, I306V*, N708D*, D759N*, A811V*, G819A*, S820T*	I699V*, E707D*, L773I*
°E21	V24A, S27G, R34Q, G66E, E100Q, K144Q, T172A, L177I, A216S, T324A, N496K, A499I, E525D, T25A*, S39T*, A401S*, D644N*	N708D*	
°U129	T25A*, S39T*, D644N*	I259L*, I306V*, N708D*, D759N*, A811V*, G819A*S820T*	I699V*, E707D*, L773I*

*Putatively novel mutations.

*No mutations found in *E. faecalis*, *E. hirae*, and *E. durans*.*

### Mobile Gene Elements (Plasmids, Insertion Sequences, Intact Prophages, and Integrons)

PlasmidFinder revealed a total of seven different plasmid associated replication genes (repUS15, repUS43, rep9c, rep9b, rep29, repUS24, repUS1). The repUS43 and repUS15 were the most common replicon types occurring in eight (66.7%) and five (41.6%) isolates, respectively (Table 1). A total of seven (58.3%) isolates had more than one plasmid replicon; however, no plasmid replicon types were detected in three isolates (one *Enterococcus feacium* and two *E. hirae*) (Table 1). There was no unique pattern concerning the replicon type, sequence type, and source of isolation. However, replicon type rep9b/c was only found in *E. faecalis* isolates with rep24 and rep29 being unique to *E. faecium* isolates.

Some ARGs were associated with insertion sequences (IS6, ISL3, and IS982), and transposons (Tn3 and Tn6000) with most of those associated with MGE being plasmid-borne (Table 3). However, the majority of ARGs were located on chromosomes and not associated with any MGEs (Supplementary Table 2). An *E. faecium* isolate (D95) from the downstream site harboured an efflux pump encoding macrolide resistance gene *msr(A)* that was associated with insertion sequence IS982. The contig carrying the *msr(A)* gene and associated MGE had very high similarity (99–100%) to a target sequence *E. faecium* HB-1 chromosome (CP040878.1) in GenBank (Table 3). An *E. faecalis* isolate (D21) from the downstream site had a plasmid-encoded trimethoprim resistance gene *dfrG* whose genetic environment had ISL3. The contig was highly similar to a target sequence in GenBank *E. faecalis* strain 133170041-3 plasmid pAD1 (CP046109.1) confirming carriage of the gene on a plasmid. Another isolate (U84) from the upstream site had a plasmid that co-carried the tetracycline resistance [*tet(M)* and *tet(L)*] and macrolide resistance *erm(B)* genes. The genetic environment of the resistance genes consisted of a recombinase and the Tn3 transposons and the contig was closely related to *E. faecalis* S7316 plasmid Ps7316optrA (LC499744.1) (Table 3). The *E. durans* isolate (E115) from the effluent site had an antibiotic resistance genetic island consisting of genes encoding resistance to aminoglycosides [*ant(6)-Ia*], chloramphenicol (*catB*), macrolides [*msr(D)* and *mef(A)*], and trimethoprim (*dfrG*). The resistance island had MGEs including several recombinases and the insertion sequence IS6 (Table 3). The resistance island was located on a contig that closely resembled a target sequence in GenBank *E. faecalis* strain transconjugant T4 plasmid pJH-T4 (KY290886.1) implying that it was located on a plasmid. The genetic environment of the tetracycline resistance gene *tet(S)* was associated with the insertion sequence IS6. Interestingly the contig carrying this *tet(S)* gene was highly similar to an *E. faecalis* strain C386 transposon Tn6000 (JN208881.1) (Table 3). The *tet(M*) resistance gene was consistently found associated with the tetracycline resistance leader peptide (*tetrLpep*) and a conjugative transposon/transfer protein (*TcpC*), genetic context *tet(M): tetrLpep: TcpC* (IN127, IN133, E21, E115, D21, U129) and the reverse context *TcpC:tetrLpep:tet(M)* in D95, D98, U84). The *TcpC* conjugative TcpC is required for efficient conjugative transfer and mediates tetracycline resistance. Notably, the genetic context was found on contigs with high similarity (98–100%) to *Enterococcus* spp. chromosomal sequences deposited in GenBank except in *E. faecalis* isolate U84 where the genes were co-carried on a plasmid with other ARGs (Table 3).

**TABLE 3:** Mobile genetic elements associated with antibiotic resistance genes in *Enterococcus* spp.

Isolate (MLST)	Contig	Synteny of resistance genes and MGE	Plasmid/chromosomal sequence with closest nucleotide homology (accession number)
***E. faecium***			
°D95/9 (ST94)	4	*Transposase:::::IS982:msr(A)*	*E. faecium* HB-1 chromosome (CP040878.1)
	39	*TcpC: tetrLpep :tet(M)*	*E. faecium* HB-1 chromosome (CP040878.1)
°D98/9 (ST94)	34	*TcpC:tetrLpep:tet(M)*	*E. faecium HB-1 chromosome (CP040878.1)*
°E21/6 (ST1096)	2	*tet(M): tetrLpep: TcpC*	*E. faecium* isolate e4456 chromosome (LR135482.1)
°U129/11 (ST361)	57	*tet(M): tetrLpep: TcpC*	*E. faecium* HB-1 chromosome (CP040878.1)
***E. faecalis***			
°IN133/11 (ST841)	3	*tet(M): tetrLpep: TcpC*	*E. faecium isolate* E0139 chromosome (LR132067.1)
°IN127/10 (ST179)	33	*tet(M): tetrLpep: TcpC*	*E. faecalis* strain HA-1 chromosome (CP040898.1)
°D21/6 (ST179)	1	ISL3:::::*dfrG*	*E. faecalis* strain 133170041-3 plasmid pAD1 (CP046109.1)
	23	*tet(M): tetrLpep: TcpC*	*E. faecalis* strain JY32 chromosome (CP045045.1)
	27	*aph(2″)-Ia::: aph(3′)-IIIa*	*E. faecalis* strain TH4125 chromosome (CP051005.1)
°U84/9 (ST300)	2	*TcpC : tetrLpep :tet(M): tet(L)::::::::::recombinase: Tn3:::: Tn3:recombinase:erm(B)*	*E. faecalis* S7316 plasmid Ps7316optrA (LC499744.1)
***E. durans***			
°E115/10	7	*tet(M): tetrLpep: TcpC*	*E. faecalis 62* chromosome (*CP022712.1*)
	22	*ant(6)-Ia::::catB::msr(D):mef(A):recombinase:recombinase:::recombinase :::IS6:::::::::::::dfrG::recombinase*	*E. faecalis* strain Transconjugant T4 plasmid pJH-T4 (KY290886.1)
	40	*tet(S)*::::IS6	*E. faecalis* strain C386 transposon Tn6000 (JN208881.1)

A total of 32 IS families were detected in the genomes (Table 1). Ten IS families occurred more than once, with the ISEfa5 (5 isolates), ISEfa11 (5), and ISEfa10 (4) being predominant. ISEfa5 and ISEfa11 occurred in the same five isolates, covering all four species (Table 1). The IS did not follow source or sequence type, albeit two *E. faecalis* isolates (IN127, D21) belonging to ST179 had the same four ISs.

Intact prophages were found within 9/12 (75%) of the genomes. Three isolates comprising one *E. faecalis* and two *E. hirae* did not possess any intact prophages. A total of seven intact prophages were identified across all the investigated isolates, with Lister_LP_101 and Entero_EFC_1 being unique to individual isolates (Table 1). The Entero_phiFL1A was the most common prophage occurring in three different isolates from the upstream, influent, and effluent sites. The Entero_phiFL3A (*n* = 2) occurred in *E. faecalis* isolates from the upstream and influent site, Lister_B025 (*n* = 2) occurred in *E. faecium* isolates from the influent and effluent sites. The occurrence of intact prophages was not according to species, as several prophages occurred in different species including Entero_phiFL1A (*E. faecalis* and *E. durans*), Lister_B025 (*E. faecium* and *E. hirae*), and Bacill_BCJA1c (*E. faecium* and *E. durans*). *E. faecalis* and *E. faecium* isolates did not have any intact prophages in common. The intact prophages did not occur according to sequence type, although *E. faecium* isolates (D95, D98) belonging to ST179 had the same prophage Entero_vB_IME197.

Seven isolates had regions encoding the T4SS type ICE, with one *E. faecium* isolate (U129) from the upstream site and the effluent isolate *E. durans* (E115) having three regions each (Table 1). The IMEs were detected in five isolates (3 *E. faecalis* and 2 *E. faecium*). Two isolates (one *E. faecalis* and one *E. faecium* isolate) harboured both the ICE and IME. *E. hirae* isolates did not harbour any of the stated MGEs except for insertion sequences implying that these might be central in horizontal gene transfer, however, none of the ARGs in these isolates were associated with MGEs. The genome of environmental *Enterococcus* spp. consists of a rich diversity of MGEs including ISs, transposons, prophages, and plasmids that probably drive genetic exchange within and among these species.

### Virulome of *Enterococcus* Isolates

A diversity of virulence genes was found in the *E. faecium* and *E. faecalis* isolates with none identified in *E. hirae* and *E. durans* (Table 4). For *E. faecalis*, a total of 20 different virulence genes were identified, including genes encoding sex pheromones, adhesion, cell invasion, aggregation, toxins, biofilm formation, cytolysin production, immunity, antiphagocytic activity, and proteases (Table 4). All the *E. faecalis* isolates had eleven of these genes (*cCF10*, *cOB1*, *cad*, *cam*E, *ace*, *SrtA*, *ebpA*, *ebpC*, *efaAfs*, *tpx*, and *gelE*) in common. In *E. faecium*, only four virulence genes were identified and included adhesins (*acm* and *efaAfm*), a sex pheromone (*cad*), and an antiphagocytic factor (*tpx*) (Table 4).

**TABLE 4:** Virulence gene profiles of environmental *Enterococcus* spp.

Isolate ID	Point of isolation	Virulence genes								
		
		Sex pheromones	Adhesion	Invasin	Aggregation	Cytolytic toxin	Biofilm formation	Antiphagocytic	Immunity	Protease
***E. faecalis***										
IN133 (ST 841)	Influent	*cCF10, cOB1, cad, camE*	*acm, ace, SrtA, efaAfs, ebpA, ebpC*	*hylB*	*agg*	*cylA, cylL, cylM*	*fsrB*	*tpx*	*ElrA*	*gelE*
IN127 (ST 179)	Influent	*cCF10, cOB1, cad, camE*	*ace, SrtA, ebpA, ebpC, efaAfs*	*hylA*	*agg*	*cylA, cylL, cylM*	*–*	*tpx*	*ElrA*	*gelE*
D21 (ST 179)	Downstream	*cCF10, cOB1, cad, camE*	*ace, SrtA, efaAfs, ebpA, ebpC*	*hylA*	*agg*	*cylA, cylL, cylM*	*–*	*tpx*	*ElrA*	*gelE*
U84 (ST 300)	Upstream	*cCF10, cOB1, cad, camE*	*ace, SrtA, ebpA, ebpC, efaAfs*	*hylB*	*agg*	*–*	*fsrB*	*tpx*	*–*	*gelE*
***E. faecium***										
IN91 (ST 361)	Influent	*–*	*Acm, efaAfm*	*–*	*–*	*–*	*–*	*–*	*–*	*–*
D95 (ST 94)	Downstream	=	*Acm, efaAfm*	*–*	*–*	*–*	*–*	*–*	*–*	*–*
D98 (ST 94)	Downstream	*–*	*acm, efaAfm*	*–*	*–*	*–*	*–*	*–*	*–*	*–*
E21 (ST 1096)	Effluent	*–*	*efaAfm*	*–*	*–*	*–*	*–*	*–*	*–*	*–*
U129 (ST 361)	Upstream	*cad*	*–*	*–*	*–*	*–*	*–*	*tpx*	*–*	*–*

*No virulence genes found in *E. hirae* and *E. duran*.*

The virulence genes in *E. faecium* were mostly devoid of any association with MGEs. Among the *E. faecalis* isolates the *gelE* (protease) was co-carried with the *fsrC* (biofilm formation) virulence gene in a genetic environment that had an integrase and IS256. This occurred in two isolates from the influent (IN127) and downstream (D21) sites with genetic context *gelE:fsrC::::integrase:::: IS256* (Table 5). The contigs bearing these virulence genes were highly similar (99 - 100%) to a chromosomal sequence in GenBank *Enterococcus faecalis* strain FDAARGOS_324 chromosome (CP028285.1) implying their carriage in the chromosome. Although, several virulence genes were found to occur together in other *E. faecalis* isolates their genetic environment did not contain any MGEs (Table 5). This implies that in addition to MGEs like ISs the transfer of virulence genes may be moderated by other processes that facilitate genetic exchange e.g., natural transformation.

**TABLE 5:** Mobile genetic elements associated with virulence genes in *E. faecalis* isolates.

Isolate	Contig	Synteny of virulence genes and MGE	Plasmid/chromosomal sequence with closest nucleotide homology (accession number)
***E. faecalis***			
IN133/11 (ST841)	13	*SrtC:ebpC:ebpB:epbA*	*Enterococcus faecalis* strain TK-P4B chromosome (CP045598.1)
	45	*CylR2:cylL:cylS*	*Enterococcus faecalis* strain FDAARGOS_528 chromosome (CP033787.1)
	48	*gelE:fsrC::fsrA*	*Enterococcus faecalis* strain L15 chromosome (CP042231.1)
IN127/10 (ST179)	8	*Cyl*:::*cyL-S:cylL-L:cylR2*	*Enterococcus faecalis* strain JY32 chromosome (CP045045.1)
	22	*gelE:fsrC::::integrase::::IS256*	*Enterococcus faecalis* strain FDAARGOS_324 chromosome (CP028285.1)
	25	*SrtC:ebpC:ebpB:epbA*	*Enterococcus faecalis* strain JY32 chromosome (CP045045.1)
D21/6 (ST179)	16	*gelE:fsrC::::integrase::::IS256*	*Enterococcus faecalis* strain FDAARGOS_324 chromosome (CP028285.1)
	30	*Cyl:::cyL-S:cylL-L:cylR2*	*Enterococcus faecalis* strain JY32 chromosome (CP045045.1)
U84/9 (ST300)	17	*ebpA:ebpB:ebpC:srtC*	*Enterococcus faecalis* strain SF28073 chromosome (CP060804.1)
	17	*gelE:fsrC::fsrA*	*Enterococcus faecalis* strain SF28073 chromosome (CP060804.1)

### MLST and Phylogenomics

MLST analysis revealed that two *E. faecalis* isolates had a common sequence type ST179; the rest of the isolates had unique sequence types, ST841, and ST300 (Table 1). The *E. faecium* genomes belonged to three sequence types, ST94 (*n* = 2), ST361 (*n* = 2), and ST1096 (*n* = 1).

**FIGURE 1:**
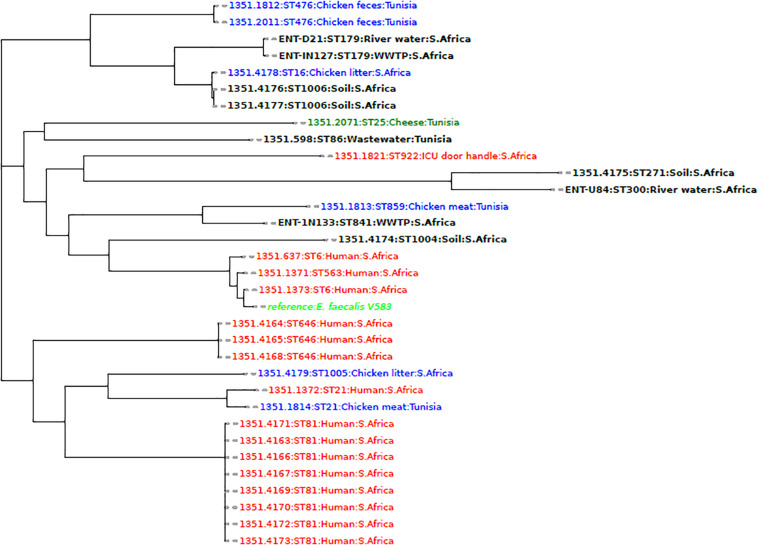
A phylogenomic tree showing the relationship of *E. faecalis* isolates from this study with African isolates from humans (red), animals (blue), and the environment (black) obtained from the PATRIC database (https://www.patricbrc.org/).

Phylogenetic analysis of the *E. faecalis* genomes from this study and those from other studies in Africa showed that the isolates were more closely related to animal and environmental isolates than to clinical isolates (Figure 1). An isolate obtained from the influent (IN133 and ST841) was more closely related to a Tunisian isolate (1351.1813, ST859) from chicken meat. An isolate (U84, ST300) from the upstream site was closely related to an isolate (1351.4175, ST271) from agricultural soil obtained in the same district of uMgungundlovu in KZN, South Africa. However, the other two isolates (D21, IN127), both ST179, clustered together and were in the same node as environmental isolates obtained from the soil and chicken litter of a sugarcane farm in KZN, South Africa (Figure 1).

**FIGURE 2:**
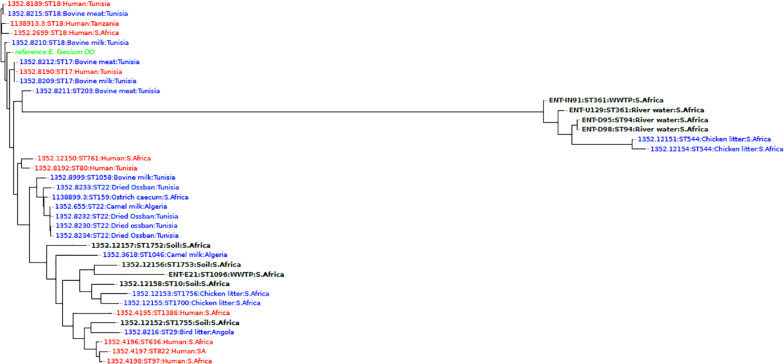
A phylogenomic tree showing the relationship of *E. faecium* isolates from this study with African isolates from humans (red), animals (blue), and the environment (black) obtained from the PATRIC database (https://www.patricbrc.org/).

Comparison of *E. faecium* genomes with other WGS isolates from Africa revealed that the isolates from this study D95, D98 (ST94), and IN91, U129 (ST361) clustered together according to sequence type, but formed a separate clade with isolates obtained from the chicken litter at a sugarcane farm in KZN, South Africa. An isolate E21 (ST1096), was found in a different clade and clustered closely with a South African soil isolate from the same farm in KZN (Figure 2).

**FIGURE 3:**
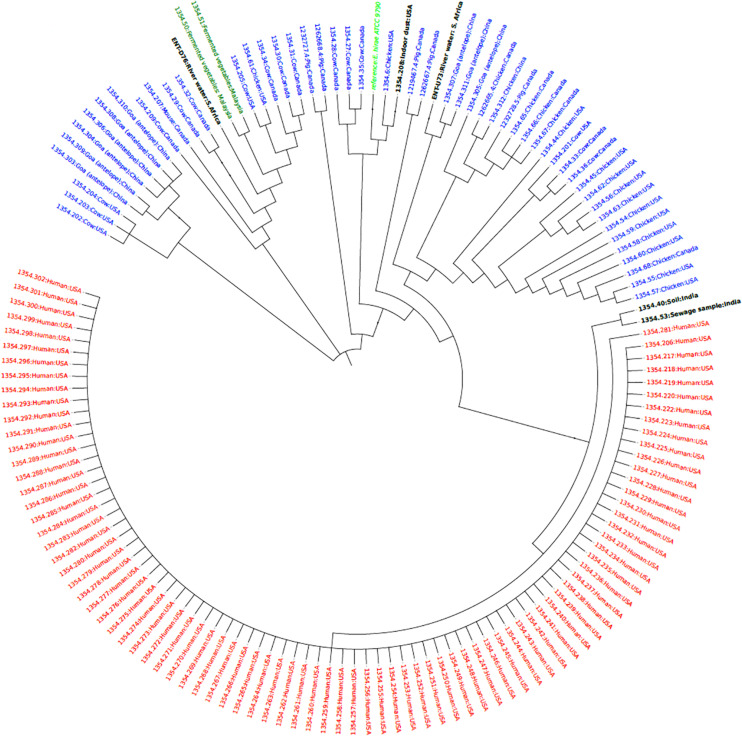
Phylogenetic tree based on SNP differences in the core genomes of *E. hirae* isolates from this study (ENT D76 and ENT U73) and other isolates from humans (red), animals (blue), environment (black) obtained from the PATRIC database (https://www.patricbrc.org/).

**FIGURE 4:**
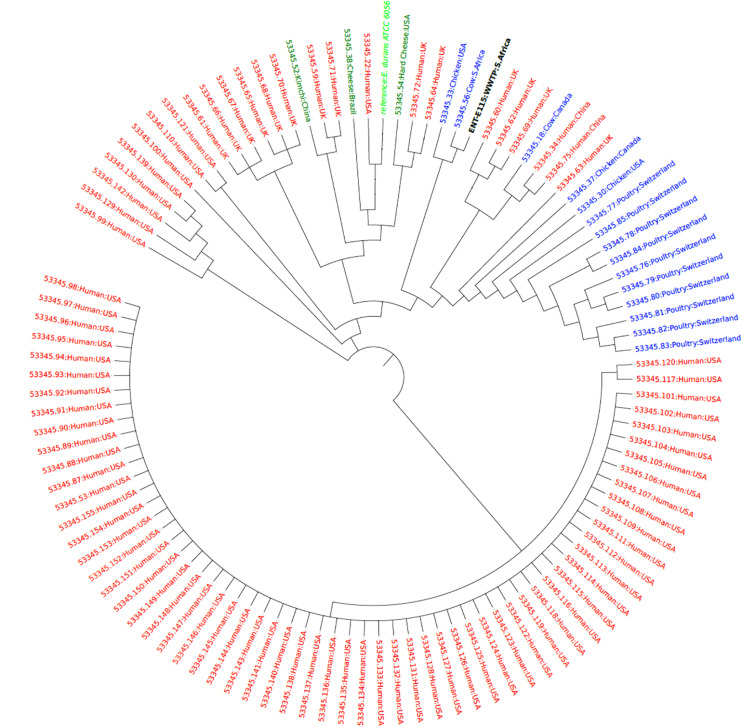
Phylogenetic tree showing the relationship of the *E. durans* isolate (black label) with other isolates obtained from humans (red), animals (blue), and food sources (green) from the PATRIC database (https://www.patricbrc.org/).

Phylogenetic analysis of *E. hirae* isolates revealed that the *E. hirae* isolates were more closely related to livestock and environmental isolates. The upstream isolate U73 was closely related and clustered closely with isolates from Goa (Tibetan antelope) fecal matter obtained in China, suggesting that the isolate could be of animal origin. The other isolate (D76) was also closely related and clustered together with isolates from fermented vegetables from Malaysia, signifying that the isolate may be from an agricultural source (Figure 3). The *E. durans* isolate clustered closely with a bovine isolate (53345.56) obtained from South Africa and an isolate frim chicken (53345.33) from the United States implying that it is an animal-associated isolate (Figure 4).

## Discussion

Bioinformatics tools were used to analyze the whole genomes of MDR *Enterococcus* isolates (*n* = 12) with similar antibiograms obtained from a WWTP and the receiving water bodies at different sampled points and at different timelines. While many ARGs were carried on plasmids, transposable elements, and insertion sequences, most were, carried on chromosomes with no association with MGEs. A few virulence genes were associated with ISs, with most occurring on chromosomes. The abundance of MGEs observed in the Enterococci genomes, however, signifies their importance in gene rearrangements and horizontal gene transfer in these environmental isolates. This study is one of the first studies to explore the resistome, virulome, mobilome, clonality, and phylogenomics of *Enterococcus* spp. obtained from the water environment in Africa.

Tetracycline resistance genes identified in this study included the *tet(M), tet*(S), and *tet(L)*. The *tet(M)* gene was present in the *E. durans* isolate and in all the *E. faecium* and *E. faecalis* isolates (Table 1). The *tet(M)* and *tet(S)* encode for ribosome protection proteins and the *tet(L)* encodes an efflux pump (Tao et al., 2010). There was a high concordance between the phenotypic AST and genotypic data with regards to TET resistance. The genetic context *tet(M):tetrLpep:TcpC* (or its reverse) was found in 9/10 (90%) of isolates that harboured the *tet(M)* gene (Table 3). The TcpC gene encodes a conjugative TcpC which is essential for efficient conjugative transfer and has previously been associated with conjugative tetracycline resistance plasmids in *Clostridium perfringens* (Bannam et al., 2006). Most of the *tet(M)* genes were located on chromosomes except for *E. faecalis* isolate U84 where the genetic context was associated with a plasmid. The genes involved in ribosome protection including *tet(M)* are typically found on both plasmids and self-conjugative transposons in chromosomes (Roberts, 1996) as evidenced in this study. Transfer of *tet(M)* in environmental Enterococci is possibly mediated by the conjugative TcpC.

Resistance to macrolides was associated mainly with the *erm(B)* and *msr(C*) genes. The *erm(B)* encodes a ribosomal methylase and is considered to be the most common gene responsible for resistance to erythromycin in enterococci; the methylase can also result in resistance to lincosamides, and streptogramin B (Miller et al., 2015). The rRNA methylases, *erm(A)*, *erm(B)*, and *erm(C)* modify specific nucleotides in the 23S rRNA and block macrolide binding (Chancey et al., 2015). Resistance to macrolides may also be caused by mutations in the 23S ribosomal RNA gene or be mediated by efflux pumps (Miller et al., 2015). All isolates that were phenotypically resistant to erythromycin had the *erm(B)* gene (Table 1). In *Enterococcus* spp. the *erm(B)* gene is considered the most widespread erythromycin resistance gene (Zaheer et al., 2020). The *msr(C*) gene which encodes an efflux pump was identified in all the *E. faecium* isolates which is consistent with earlier studies that stated that the gene seems to be specific for this species (Zaheer et al., 2020). The genome of the *E. durans* isolate had the efflux pump encoding genes *msr(D)* and *mef(A)* which were unique to this isolate. The macrolide efflux (*mef*) genes were initially identified in *Streptococcus pyogenes* (Sutcliffe et al., 1996) and *S. pneumoniae* (Gay and Stephens, 2001) and have been noted to always occur upstream and to be co-transcribed with an ATP-binding subunit ABC-transporter gene *msr(D)*, functioning as a dual efflux pump (Ambrose et al., 2005). The genes were located on a resistance island consisting of MGEs (recombinase, IS6) together with chloramphenicol, aminoglycoside, and trimethoprim resistance genes (Table 3). There is a possibility that this resistance island is transmissible within and across sub-species, although its transferability was not experimentally investigated. Although *E. durans* strains rarely cause infection, the occurrence of these resistance genes implies the importance of these organisms as environmental reservoirs which could potentially mediate the transfer of these genes to pathogens of clinical or veterinary importance.

Enterococci are inherently resistant (low-level) to aminoglycosides, mostly due to the presence of the *aac(6*′*)-Ii* gene. Some isolates, however, exhibit high-level resistance to gentamicin and streptomycin and are clinically important (Sanderson et al., 2020). The presence of other acquired aminoglycoside resistance genes including *aac*(6′)-Ie–*aph*(2″)-Ia, *aph*(3′)-IIIa, and *ant*(6)-*Ia* confers high-level resistance to various aminoglycosides (Said et al., 2015). Except for *E. durans* isolate E115 none of the aminoglycoside resistance genes were associated with MGEs and most were borne on the chromosome (Table 3). Isolate E115 had the *ant*(6)-*Ia* gene which formed part of a resistance island that was on a plasmid. The isolate exhibited high-level resistance to streptomycin (Table 1).

A diversity of virulence genes was identified in the genomes of the sequenced *E. faecalis* and *E. faecium* isolates (Table 4). The *gelE* and *fsr* genes have been shown to occur together in *E. faecalis* isolates from healthy and sick animals (Šeputiene et al., 2012). The *fsr*ABDC operon has been shown to regulate the expression of the *gelE* gene and other virulence genes (Hancock and Perego, 2004). The *gelE* encodes an extracellular zinc endopeptidase that cleaves a broad range of substrates including collagen and gelatin. It accentuates the pathogenesis of endocarditis caused by *E. faecalis* (Thurlow et al., 2010). The *gelE* and *fsr* genes occurred together in several *E. faecalis* isolates including IN133 and U84 that had genetic context *gelE:fsrC::fsrA* (Table 5). Isolates D21 and IN127 both had genetic context *gelE:fsrC::::integrase:::: IS256* suggesting that IS256 plays a role in the transmission of these virulence genes. The IS256 is prevalent in the genomes of MDR enterococci and staphylococci where it occurs either independently or is associated with ARGs or virulence genes involved in biofilm formation (Hennig and Ziebuhr, 2010; Kim et al., 2019). Other virulence genes were not associated with MGEs suggesting that processes like natural transformation may be important in the transfer of these genes. Generally, the repertoire of virulence genes revealed in this study point to the presence of potentially pathogenic *Enterococcus* spp. in the investigated water environment.

MLST revealed distinct sequence types that are associated with clinical, animal, and agricultural sources. For *E. faecalis* isolates, ST179 was the most common sequence type (*n* = 2) a finding similar to other studies (Zaheer et al., 2020). For the *E. faecium* isolates, ST94 (*n* = 2) and ST361 (*n* = 2) were the most prevalent sequence types. In the study by Zaheer et al. (2020) the ST94 was the most abundant ST in the cattle feedlot catch basin and was found in other sources namely, surface water, urban wastewater, and from the clinic. This possibly points to the ubiquitous nature of this sequence type. The ST361 is not one of the notable *E. faecium* STs as it has not been implicated in clinical cases which are mostly attributed to the ST17, ST18 and, ST78 lineages (Palmer et al., 2014). A study from the United Kingdom used WGS to investigate the prevalence of vancomycin-resistant *E. faecium* in 20 WWTPs and reported an *E. faecium* ST361 from a WWTP (Gouliouris et al., 2018).

The phylogenomics of *E. faecalis* and *E. faecium* isolates revealed that all isolates were closely related to environmental or animal isolates, and not clinical isolates (Figures 1, 2). However, the *E. faecalis* influent isolate (IN133, ST841) harbored the cytolysin genes that have been attributed to clinical *E. faecalis* isolates intimating pathogenic potential (Zaheer et al., 2020). Phylogenetic analysis of the *E. hirae* isolates in this study revealed a close association with other animal and environmental isolates (Figure 3). *E. hirae* is known to inhabit a variety of animals and plants (Byappanahalli et al., 2012) and has been widely associated with cattle feces, chicken broilers, and associated production systems (Rehman et al., 2018; Zaheer et al., 2020). The *E. durans* isolate was closely related to animal isolates (Figure 4), indicating that it may be of animal origin. *E. durans* isolates are known to inhabit humans, animals, and insects and occasionally cause human infections (Byappanahalli et al., 2012). The isolate investigated in this study lacked virulence determinants and is most likely a potential reservoir of ARGs. Although a small subset of *Enterococcus* spp. isolates were used, this study adds to the limited knowledge of the resistome, virulome, mobilome, and phylogenies in environmental Enterococci in Africa. Future studies should look to use a larger sample size and greater diversity of *Enterococcus* spp. from diverse geographical locations.

## Conclusion

This is the first report of genomic diversity of *Enterococcus* spp. found in wastewater and associated river water in KwaZulu-Natal, South Africa. *Enterococcus* spp. showed a rich repertoire of ARGs and virulence factors implying that the water environment is a substantive reservoir of MDR microbes which are potential pathogens. Genomic analysis of the Enterococci isolates allowed for the description of the resistome, virulome, and mobilome as well as the determination of phylogenetic relationships with animal, agricultural and environmental isolates. Such work allows a deeper understanding of the potential transmission dynamics related to the spread of antibiotic resistance in the water environment.

## Data Availability Statement

The datasets presented in this study can be found in online repositories. The names of the repository/repositories and accession number(s) can be found in the article/Supplementary material.

## Author Contributions

SE, JM, AA, and DA: co-conceptualized the study. JM: performed the experiments and wrote the manuscript. JM, AA, and DA: analyzed the data. SE, AA, and DA: supervision. SE: funding acquisition. All authors undertook critical revision of the manuscript and reviewed, edited, and approved the final manuscript.

## Disclaimer

Any opinion, finding, and conclusion or recommendation expressed in this material is that of the authors, and neither the NRF nor the other funding bodies accept any liability in this regard.

## Conflict of Interest

SE is chairperson of the Global Respiratory Infection Partnership and a member of the Global Hygiene Council, funded by unrestricted educational grants from Reckitt and Benckiser (Pty.), United Kingdom. The remaining authors declare that the research was conducted in the absence of any commercial or financial relationships that could be construed as a potential conflict of interest.

## Footnotes

1 https://github.com/najoshi/sickle

2 http://cge.cbs.dtu.dk/services/MLST/

3 https://cge.cbs.dtu.dk/services/ResFinder/

4 https://cge.cbs.dtu.dk/services/PlasmidFinder/

5 https://cge.cbs.dtu.dk/services/VirulenceFinder/

6 https://isfinder.biotoul.fr/

7 https://phaster.ca/

8 http://db-mml.sjtu.edu.cn/ICEberg/

9 http://rast.nmpdr.org/seedviewer.cgi

10 https://www.geneious.com

11 https://www.patricbrc.org/

12 https://cge.cbs.dtu.dk/services/CSIPhylogeny/

13 https://itol.embl.de/

## Supplementary Material

Supplementary Table 1 Genome and assembly characteristics of sequenced *Enterococcus* spp. isolates from a wastewater treatment plant and its associated waters.

Supplementary Table 2 Antibiotic resistance genes not associated with any mobile genetic elements in enterococci.
