# Supplementary material for: Genomic Analysis of Enterococcus spp. Isolated From a Wastewater Treatment Plant and Its Associated Waters in Umgungundlovu District, South Africa
Source: Front Microbiol. 2021 Jun 14;12:648454. doi: 10.3389/fmicb.2021.648454 (PMC8236953; doi:10.3389/fmicb.2021.648454)
Supplement: Supplementary Table 1 — Genome and assembly characteristics of sequenced Enterococcus spp. isolates from a wastewater treatment plant and its associated waters. [file Data_Sheet_1.docx]

**Table S1**. Genome and assembly characteristics of sequenced *Enterococcus* spp. isolates from a wastewater treatment plant and its associated waters.

| **Isolate ID** | **Species** | **Source** | **Sequence length (bp)** | **No. of contigs** | **GC content (%)** | **Longest contig size (bp)** | **N50 value (bp)** | **L50 value** |
| --- | --- | --- | --- | --- | --- | --- | --- | --- |
| INF133/11 | *E. faecalis* | Influent | 3202121 | 51 | 37.0 | 496589 | 385477 | 4 |
| INF127/10 | *E. faecalis* | Influent | 2948602 | 33 | 37.3 | 624804 | 407232 | 3 |
| D21/6 | *E. faecalis* | Downstream | 2991631 | 34 | 37.3 | 527503 | 350389 | 4 |
| U84/9 | *E. faecalis* | Upstream | 2902616 | 17 | 37.2 | 829991 | 356305 | 3 |
| INF91/9 | *E. faecium* | Influent | 3225610 | 718 | 38.0 | 133398 | 31215 | 25 |
| E21/6 | *E. faecium* | Effluent | 2531743 | 93 | 38.1 | 198433 | 73574 | 11 |
| D95/9 | *E. faecium* | Downstream | 2562575 | 40 | 38.4 | 246994 | 186235 | 6 |
| D98/9 | *E. faecium* | Downstream | 2564395 | 36 | 38.4 | 246994 | 193163 | 6 |
| U129/11 | *E. faecium* | Upstream | 2794367 | 59 | 38.0 | 233957 | 106058 | 8 |
| D76/8 | *E. hirae* | Downstream | 2674477 | 12 | 36.9 | 558569 | 283640 | 3 |
| U73/8 | *E. hirae* | Upstream | 2925170 | 14 | 36.6 | 1266460 | 527524 | 2 |
| E115/10 | *E. durans* | Effluent | 3246276 | 115 | 37.6 | 423641 | 211603 | 7 |

Key: N50 = smallest contig of the size-sorted contigs that make up at least 50% of the respective assembly

L50 = number of contigs that make up at least 50% of the respective total assembly length

**Table S2**: Antibiotic resistance genes not associated with any mobile genetic elements in Enterococci

| **Isolate (MLST)** | **Contig** | **Synteny of resistance genes and MGE** | **Plasmid/chromosomal sequence with closest nucleotide homology (accession number)** |
| --- | --- | --- | --- |
| ***E. faecium*** | | | |
| INF91/9 (ST361) | 6 | *tet(M): tetrLpep* | *E. faecium* HB-1 chromosome (CP040878.1) |
|  | 8 | *msr(C)* | *E. faecium* HB-1 chromosome (CP040878.1) |
|  | 28 | *aa6 (‘) -li* | *E. faecium* HB-1 chromosome (CP040878.1) |
| D95/9(ST94) | 36 | *aa6 (‘) -li* | *E. faecium* HB-1 chromosome (CP040878.1) |
| D98/9(ST94) | 31 | *aa6 (‘) -li* | *E. faecium* HB-1 chromosome (CP040878.1) |
|  | 33 | *msr(C)* | *E. faecium* strain DMEA02 chromosome *(CP043484.1)* |
| E21/6(ST1096) | 2 | *Msr(C)* | *E. faecium* isolate e4456 chromosome (LR135482.1) |
|  | 4 | *aa6 (‘) –li* | *E. faecium* isolate E9101 chromosome (LR135474.1) |
| U129/11(ST361) | 41 | *aa6 (‘) –li* | *E. faecium* HB-1 chromosome (CP040878.1) |
|  | 42 | *Msr(C)* | *E. faecium* HB-1 chromosome (CP040878.1) |
| ***E. faecalis*** | | | |
| IN127/10(ST179) | 27 | *erm(B)* | *E. faecalis* strain HA-1 chromosome (CP040898.1) |
| D139/11(ST1) | 14 | Isa(A) | *E. faecalis* OG1RF (CP002621.1) |
| D21/6(ST179) | 8 | dfrE | *E. faecalis* strain JY32 chromosome (CP045045.1) |
|  | 27 | *aph(2'')-Ia:::* *aph(3')-IIIa:* | *E. faecalis* strain TH4125 chromosome (CP051005.1) |
|  | 34 | *erm(B)* | *E. faecalis* strain TH4125 chromosome (CP051005.1) |
| U84/9(ST300) |  |  |  |
|  | 3 | *erm(B)* | *E. faecalis* strain SF28073 chromosome (CP060804.1) |
|  | 13 | *dfrG* | *E. faecalis* strain SF28073 chromosome (CP060804.1) |
|  | 17 | *dfrE* | *E. faecalis* strain SF28073 chromosome (CP060804.1) |
| ***E. hirae*** | | | |
| INF5/5 | 17 | *aac(6')-Ii* | *E. faecalis strain* 4928STDY7387713 chromosome (LR607361.1) |
| D76/8 | 12 | *aac(6')-Iid* | *E. faecalis strain* 4928STDY7387713 chromosome (LR607361.1) |
| U71/8 | 5 | *aac(6')-Ii* | *E. faecalis strain* *4928STDY7387713 chromosome (LR607361.1)* |
| U73/8 | 5 | *aac(6')-Ii* | *E. faecalis strain* 4928STDY7387713 chromosome (LR607361.1) |
| ***E. durans*** | | | |
| E115/10 | 4 | *aac(6')-Ii* | *E. faecalis strain KB1 (CP022712.1)* |
